# Development of a framework for genotyping bovine-derived *Cryptosporidium parvum*, using a multilocus fragment typing tool

**DOI:** 10.1186/s13071-015-1107-8

**Published:** 2015-10-01

**Authors:** Emily J. Hotchkiss, Janice A. Gilray, Marnie L. Brennan, Robert M. Christley, Liam J. Morrison, Nicholas N. Jonsson, Elizabeth A. Innes, Frank Katzer

**Affiliations:** Moredun Research Institute, Pentlands Science Park, Bush Loan, Penicuik, Edinburgh EH26 0PZ UK; School of Veterinary Medicine and Science, University of Nottingham, Sutton Bonington Campus, Loughborough, LE12 5RD UK; Institute of Infection and Global Health, University of Liverpool, NIHR Health Protection Research Unit in Emerging and Zoonotic Infections, Leahurst Campus CH64 7TE, Liverpool, L69 7BE UK; Roslin Institute, Royal (Dick) School of Veterinary Studies, University of Edinburgh, Easter Bush, Midlothian, EH25 9RG UK; Institute of Biodiversity, Animal Health and Comparative Medicine, University of Glasgow, Bearsden Road, Glasgow, G61 1QH UK

**Keywords:** *Cryptosporidium parvum*, Multilocus, Genotyping, Bovine

## Abstract

**Background:**

There is a need for an integrated genotyping approach for *C. parvum*; no sufficiently discriminatory scheme to date has been fully validated or widely adopted by veterinary or public health researchers. Multilocus fragment typing (MLFT) can provide good differentiation and is relatively quick and cheap to perform. A MLFT tool was assessed in terms of its typeability, specificity, precision (repeatability and reproducibility), accuracy and ability to genotypically discriminate bovine-derived *Cryptosporidium parvum*.

**Methods:**

With the aim of working towards a consensus, six markers were selected for inclusion based on their successful application in previous studies: MM5, MM18, MM19, TP14, MS1 and MS9. Alleles were assigned according to the fragment sizes of repeat regions amplified, as determined by capillary electrophoresis. In addition, a region of the GP60 gene was amplified and sequenced to determine *gp60* subtype and this was added to the allelic profiles of the 6 markers to determine the multilocus genotype (MLG). The MLFT tool was applied to 140 *C. parvum* samples collected in two cross-sectional studies of UK calves, conducted in Cheshire in 2004 (principally dairy animals) and Aberdeenshire/Caithness in 2011 (beef animals).

**Results:**

Typeability was 84 %. The primers did not amplify tested non-*parvum* species frequently detected in cattle. In terms of repeatability, within- and between-run fragment sizes showed little variability. Between laboratories, fragment sizes differed but allele calling was reproducible. The MLFT had good discriminatory ability (Simpson’s Index of Diversity, SID, was 0.92), compared to *gp60* sequencing alone (SID 0.44). Some markers were more informative than others, with MS1 and MS9 proving monoallelic in tested samples.

**Conclusions:**

Further inter-laboratory trials are now warranted with the inclusion of human-derived *C. parvum* samples, allowing progress towards an integrated, standardised typing scheme to enable source attribution and to determine the role of livestock in future outbreaks of human *C. parvum*.

## Background

The protozoan parasite *Cryptosporidium* is an enteropathogen of the Apicomplexa phylum, with 26 defined species and more than 60 genotypes which have not been assigned species status [1]. Some of these species/genotypes are strongly host associated (*C. hominis*, *C. bovis*) whereas others are capable of infecting and causing disease in a wide range of host species. *C. parvum* is prevalent worldwide and infects both humans and animals, with serious clinical outcome in some populations. Neonatal livestock have been shown to shed this species in large numbers [2, 3] and are thought to be the most important zoonotic source of infection for humans; in the UK, it is estimated that almost all dairy and beef farms are infected and longitudinal studies suggest that approximately 100 % of all calves will shed the oocysts at some point in the first few weeks of life (Thomson, unpublished data). Zoonotic and anthroponotic transmission can occur directly, via contact with shedding individuals, or indirectly, with the highly resistant oocysts being ingested via drinking or recreational water or food.

The different species of *Cryptosporidium* cannot be distinguished by microscopy therefore molecular tools are invaluable in assigning species and determining zoonotic potential. Many laboratories subtype within *C. parvum* and *C. hominis* by sequence analysis of the polyserine tract in the GP60 gene, which has variable numbers of TCA and TCG codons between subtypes [4]. Global epidemiological studies have utilised this typing tool to identify host substructuring within *C. parvum*, as allele IIc [5], for example, has only been found to date in humans.

At a national or regional level, it is important to determine subtypes of a species to aid in source attribution in the event of human infection. In this case, sequencing of the GP60 gene alone is unlikely to provide sufficient levels of discrimination, particularly where common subtypes exist and circulate widely within potential source populations, for example *gp60* subtype IIaA15G2R1, which is prevalent worldwide in both humans and cattle [6, 7]. To enable rapid investigation in the event of an outbreak of cryptosporidiosis in humans, harmonised methods of molecular typing must be adopted by both public health agencies and workers in the veterinary field. Knowledge of genotypes circulating in potential source populations, such as livestock and wildlife, would be invaluable. However, whether these baseline data are available or not, the ability to easily, robustly and rapidly screen potential sources in real time is an essential requirement of any tool to be used within an outbreak situation.

With time, the field of genomics may provide the gold standard in discriminatory typing. However genomics within the field of *Cryptosporidium* has been lagging behind other genera, possibly due to the lack of an *in vitro* culture system and issues with the low GC composition and poly T/A runs; only 3 published genomes are currently available. Much of the work to date at the genome level has focused on differences between *Cryptosporidium* species, with a current paucity of data on within-species comparisons.

Multilocus fragment typing (MLFT) uses tandem repeat units within the genome, often called micro- or mini-satellites, within coding or non-coding regions. Mini/micro-satellite regions have been shown to provide good differentiation in moderately conserved species. Multiple regions, or markers, are amplified by PCR and the resulting amplicons are sized by gel or capillary electrophoresis (CE). Length polymorphisms due to variable numbers of nucleotide repeats are the basis for genotyping and the alleles at the different markers are combined to give a multilocus genotype (MLG). Using CE, one primer is given a fluorescent label to allow sizing. Fragment sizing is preferable to sequence analysis as this can often be challenging across repeat regions. Another advantage of MLFT over multilocus sequence typing (MLST), as trialled primarily with *C. hominis* [8–10], is that samples containing mixed genotypes are readily identified by secondary peaks on trace files. Studies have demonstrated that mixed genotypes occur in cattle faeces at variable rates from 0.8 to 37 % depending on the geographic region [11, 12]. In addition, fragment sizing is rapid and cost-effective, which may be important in an outbreak situation. Whereas the use of MLFT within a laboratory may be informative, technical difficulties arise when comparing results between laboratories. It has been shown that the observed sizes depend on more than the actual length of the fragments amplified; other potential factors include sequence composition and DNA conformation, the machine used and running conditions [13, 14].

A recent review of the use of MLFT in published *Cryptosporidium* research has highlighted the lack of an integrated approach or validation of this, potentially valuable, typing tool [15]. In the current study we used markers which appeared to be informative from previous studies in order to move towards a consensus for *C. parvum*. We applied this tool to bovine *C. parvum* samples and report on the performance of the tool in terms of sensitivity (typeability), specificity, discriminatory ability, repeatability and reproducibility.

## Methods

### Study population

Faecal sample*s* from two cross-sectional studies of UK calves were processed by standard methods [7, 16]. Briefly, approximately 200 μg of faeces was placed in lysis buffer and subjected to freeze-thaw cycles before being processed by QIAamp DNA Stool Minikit (Qiagen). Those confirmed as *C. parvum* (*n* = 140) by sequence analysis of the 18S rRNA locus [17] were further characterised by MLFT. The *C. parvum* samples were sourced from: a) 92 calves from 21 beef farms in Aberdeenshire and Caithness, Northeast Scotland, collected in Spring 2011, and b) 48 calves from 21 (predominantly dairy) farms, Cheshire, Northwest England, collected in Spring 2004 [7, 16].

### Markers

A panel of 6 markers was selected based on previous studies: MM5, MM18, MM19, TP14, MS1 and MS9 (Table 1). In addition, a region of the GP60 gene was amplified as previously described [7] and sequenced to provide *gp60* subtype, which was added to the allelic profile to assign multilocus genotypes (MLGs). External primers were designed to those in the literature for use in the first round of a nested PCR reaction (Table 2) [18]. FAM or HEX was used to 5′ label one of the second round primers (MWG Eurofins).

**Table 1:** Markers selected for multilocus fragment typing of cattle-derived *Cryptosporidium parvum*

Marker	Chromosome	Repeat	Reference
MM5	6	TC(T/C)	[12]
MM18	8	(C/G)CAG(A/G)A	[12]
MM19	8	GGAGCT	[12]
TP14	8	CAA	[20]
MS1	2	GG(C/T)GG(T/A)ATGCCA	[33]
MS9	5	TGGATC	[19]
*gp60* ^a^	6	TC(A/G)	[4]

^a^ *gp60* was subjected to sequence analysis

**Table 2:** Primers used in nested PCR reactions in multilocus fragment typing of cattle-derived *Cryptosporidium parvum*

Marker		Forward	Reverse	Reference
MM5	1°	TCACAAGTTACCCCTTCTGATGCTG	TCCACCTCCGGATTGGTTGTG	[18]
	2°	CCTGGACTTGGATTTGGACTTACACC	GGAGAAGATAAGCTAGCCGAATCT	[12]
MM18	1°	GTTCAGCTGATACGGGTTTGCAACA	CATCACCATCTCCTCCGCCAGA	[18]
	2°	CTTTCTGGAGGGTTTGTTCCTCC	CTTCCTGATGATCCAGGCCAAGC	[12]
MM19	1°	TGGTTTTAGCTAAGGAAGCGATAG	CTGCTGCTGCTGTTGCTTTA	[18]
	2°	GATTCTGTCAACTTTGAATTCAG	CCAACCCCGAATTCATTTCCAAC	[12]
TP14	1°	GAGAAGGAGCAATGGGAGCA	TCCTCCTTTTTGCCCTTGAA	[18]
	2°	CTAACGTTCACAGCCAACAGTACC	CAATAAAGACCATTATTACCACC	[20]
MS1	1°	AAGGGTGAGGATGAGCAGAA	TTCTTAACTTTCCATTTTGAGTGA	Current study
	2°	TTAGTCGACCTCTTCAACAGTTGG	GGAACACCATCCAAGAACCAAAGGT	[26]
MS9	1°	TTAGTCGACCTCTTCAACAGTTGG	CAGAAT TGGAATCATTTTCTGAAT	Current study
	2°	GGACTAGAAATAGAGCTTTGGCTGG	GTCTGAGACAGAATCTAGGATCTAC	[19]
*gp60*	1°	ATAGTCTCCGCTGTATTC	GAGATATATCTTGGTGCG	[7]
	2°	TCCGCTGTATTCTCAGCC	CGAACCACATTACAAATGAAG	[7]

### PCR

Nested PCR was carried out. Mastermix consisted of 10X PCR buffer which was prepared in house using 45 mM Tris–HCl (Trizma base, Sigma; HCl: Fisher Scientific), 11 mM (NH_4_)_2_SO_4_, 4.5 mM MgCl_2_, 0.113 mg/ml BSA, 4.4 μM EDTA (all Sigma) and 1.0 mM dATP, dATC, dGTP, dTTP (VH Bio Ltd). One unit of Biotaq (Bioline), 20 pmol primer and 1ul DNA template was added in a 20ul reaction volume. PCR reactions were performed in a G-Storm thermocycler (Gene Technologies Ltd) and cycling conditions were 30 cycles of 95 °C for 50s, 50 °C for 50s and 65 °C for 60s. PCR product from the primary reaction was diluted 1:100 before use as template in the second round PCR.

### Fragment analysis

Labelled PCR products were subjected to fragment analysis using capillary electrophoresis (CE) via ABI 3730 (Applied Biosystems; University of Dundee), using size standard Genescan ROX500 (Applied Biosystems). A subset of samples was also re-sized using ROX400HD (Applied Biosystems). Trace files were then analysed using both Peak Scanner (Applied Biosystems) and STRand (https://www.vgl.ucdavis.edu/informatics/strand.php). Secondary peaks, representing mixed genotype infections, were assigned when their height was ≥ 0.33 of the primary peak and where the size of the secondary fragment had been detected in other isolates as a primary peak.

### Typeability and specificity

Typeability was assessed for each marker as well as for the combined MLFT/*gp60* tool; it was calculated as the number of samples which were successfully assigned an allele number or MLG divided by the total number of samples trialled. PCRs were repeated for samples which did not produce an amplicon at one or more markers.

In order to verify specificity of the primers (i.e., their ability to exclusively amplify the target DNA), a selection of non-*C. parvum* species likely to be present in cattle samples were included: *C. bovis* (*n* = 3), *C. ryanae* (*n* = 1) and *C. andersoni* (*n* = 1). Samples were single-species infections as determined by multiplex PCR (data not shown) and confirmed by sequence analysis of the 18S rRNA gene. These samples were processed with MLFT primers in triplicate PCR reactions. In addition, specificity was tested using primer-BLAST to ascertain whether the primers aligned with sequences deposited in GenBank and were therefore likely to amplify other organisms or non-*parvum Cryptosporidium* species.

### Precision: repeatability and reproducibility

In order to determine repeatability of fragment sizing, a subset of the samples was amplified and sized on several different occasions and fragment sizes compared between runs (between-run variation). In addition, the same sample was repeated within a PCR plate and sized within a run (within-run variation). A representative of each known allele was included in every PCR run and sizing plate.

To assess reproducibility, bovine-derived *C. parvum* DNA was made available which had previously been amplified and sized in another laboratory [12, 19, 20]. These samples were processed in our laboratory to assess the effect of machine and running conditions on the reproducibility of both fragment sizing and allele assignation.

### Discriminatory ability

Simpson’s Index of Diversity (SID) was used to assess discriminatory ability. This value provides an estimate of the probability that two epidemiologically-unrelated samples selected at random will be different alleles/genotypes [21]. SID with 95 % confidence interval was calculated using V-DICE (Variable Number Tandem Repeat Diversity and Confidence Extractor; http://www.hpa-bioinformatics.org.uk/cgi-bin/DICI/DICI.pl) for differentiation of *C. parvum* into a) different alleles for each marker, b) MLGs using MLFT and c) *gp60* subtypes using sequence analysis of *gp60* alone. Where mixed profiles were obtained, only the primary peak was used to assign alleles for this analysis.

## Results

### Typeability and specificity

Within the Scottish survey, 92 samples were *C. parvum*-positive and of these, 2 samples failed to amplify with *gp60* primers and one amplicon gave a mixed *gp60* profile. Within Cheshire samples 1/48 samples gave a mixed *gp60* profile and therefore we were unable to conclusively assign a *gp60* subtype. Overall therefore the typeability of *gp60* sequencing was 136/140 or 0.97, with 95 % confidence interval (CI) of 0.93–0.99 (Table 3).

**Table 3:** Multilocus fragment typing of *Cryptosporidium parvum* samples sourced from 118 UK calves

	MM5	MM18	MM19	TP14	MS1	MS9	*gp60*	MLG
	235	288	298	296	361	455	IIaA15G2R1	1
	262	288	298	304	361	455	IIaA15G2R1	2
	235	288	298	296	361	455	IIaA17G1R1	3
	262	288	304	296	361	455	IIaA15G2R1	4
	262	294	292	304	361	455	IIaA15G2R1	5
	262	288	292	296	361	455	IIaA15G2R1	6
	262	294	292	304	361	455	IIaA17G1R1	7
	235	288	292	296	361	455	IIaA18G3R1	8
	262	294	298	296	361	455	IIaA17G1R1	9
	262	294	298	304	361	455	IIaA15G2R1	10
	262	288	292	304	361	455	IIaA15G2R1	11
	262	288	298	296	361	455	IIaA15G2R1	12
	235	288	270	296	361	455	IIaA15G2R1	13
	262	288	316	296	361	455	IIaA15G2R1	14
	262	288	292	296	361	455	IIaA17G1R1	15
	262	288	253	296	361	455	IIaA15G2R1	16
	262	288	298	296	361	455	IIaA16G3R1	17
	262	288	298	296	361	455	IIaA19G1R1	18
	262	318	298	296	361	455	IIaA15G2R1	19
	262	288	298	296	361	455	IIaA17G1R1	20
	262	294	281	296	361	455	IIaA15G2R1	21
	262	294	298	296	361	455	IIaA15G2R1	22
	262	294	292	296	361	455	IIaA18G1R1	23
Typeability	0.91	0.92	0.91	0.90	0.93	0.96	0.97	0.84
(95 % CI)	(0.85–0.95)	(0.86–0.96)	(0.86–0.95)	(0.84–0.94)	(0.87–0.96)	(0.90–0.99)	(0.93–0.99)	(0.77–0.90)
SID (95 % CI)	0.35 (0.26–0.43)	0.41 (0.32–0.50)	0.60 (0.54–0.67)	0.35 (0.26–0.43)	0.00 (0.00–0.06)	0.00 (0.00–0.06)	0.44 (0.33–0.54)	0.92 (0.90–0.94)

*gp60* subgenotypes are included in multilocus genotype (MLG) assignment. Typeability and Simpson’s Index of Diversity (SID) are reported for allele and MLG assignment, with 95 % confidence intervals (95 % CI)

All 92 Scottish *C. parvum* samples were subjected to MLFT at 6 markers. Of these, 82 were successfully assigned an allele at all 6 markers. Within Cheshire samples, there was insufficient DNA template resulting in some missing data for markers MS1 (*n* = 5) and MS9 (*n* = 26). Typeability for these markers was calculated using adjusted denominators (Table 3). 38/48 Cheshire samples were successfully assigned an allele at the 4 remaining markers. Samples occasionally failed to amplify at one or more loci, despite repeated attempts. Of these, 7/20 failed to amplify at any of the tested loci.

Overall, excluding MS1 and MS9 due to missing data, 118/140 samples were typed with the combined scheme of 4 MLFT markers and *gp60* sequencing, resulting in typeability of 0.84 (95 % CI 0.77–0.90) (Table 3). No marker was significantly different in terms of typeability, ranging from 0.90 (TP14) to 0.96 (MS9) (Table 3).

None of the non-*C. parvum* samples amplified with any of the primers. Primer-BLAST results indicated that *C. hominis* would be co-amplified by these primers, with 100 % match for MM18 primers with *C. hominis* accession number XM_661101, 1 base difference in MM5_reverse and MS1_reverse when aligned with *C. hominis* sequences (XM_661101 and XM_661662) and 4 bases differing in MS9_forward and *C. hominis* XM_660477. For MM19, both the *C. parvum* and *C. hominis* reference genomes (XM_627368 and XM_660787) were missing base 8 of our forward primer sequence, but the reverse primer aligned with both species 100 %. No other *Cryptosporidium* spp. aligned with these primers.

### Precision: repeatability and reproducibility

In terms of repeatability, within-run variation was assessed as the range of fragment sizes obtained when the same DNA sample was amplified several times within a PCR reaction. In all cases variation was negligible (<1 bp). Between-run variation was assessed as the range in sizes obtained when the same DNA was amplified in different PCR reactions. Across all markers the median range of sizes produced was minimal at 0.4 bp (Table 4), although MM5 allele 2 had a lower repeatability with a range of 1.9 bp in seven replicates. Allele calling was straightforward however by comparing with sizes of the known alleles which were included in each run as controls.

**Table 4:** Mean fragment sizes of *Cryptosporidium parvum* DNA samples amplified in multiple separate PCR reactions

Marker	Allele	Mean Size (bp)	SE Mean (bp)	Range (bp)	N
MM5	1	262.5	0.08	0.6	7
	2	235.3	0.25	1.9	7
MM18	1	288.3	0.04	0.4	9
	2	293.9	0.05	0.3	8
	3	318.1	0.05	0.3	5
MM19	1	297.7	0.04	0.3	8
	2	303.8	0.04	0.3	9
	3	292.1	0.06	0.5	8
	4	316.4	0.09	0.3	3
	5	269.8	0.11	0.6	5
	6	252.8	0.10	0.6	6
	7	281.1	0.07	0.4	5
TP14	1	295.9	0.12	0.7	6
	2	304.7	0.14	0.8	6
MS1	1	361.2	0.06	0.4	6
MS9	1	455.1	0.04	0.3	6

*N* number of replicates. Median range = 0.4 bp

For reproducibility, sizes were shown to differ between laboratories, generally within the range of 1–2 bp (Table 5). The major difference was in marker MS9 where all alleles differed in measured size by a consistent 11 bp; this difference was maintained after repeating the amplification and sizing. Allele assignment was concordant.

**Table 5:** Comparison of fragment sizes obtained when bovine *Cryptosporidium parvum* DNA was processed in two different laboratories

Marker	MRI allele	MRI		UoG	
		Size (bp)	Prevalence (*n* = 118)	Size (bp)^a^	Prevalence (*n* = 211)
MM5	1	262	78 %	260	69 %
	2	235	22 %	233	29 %
	NA	288	0 %	287	0.5 %
MM18	1	288	73 %	290	56 %
	2	294	22 %	296	2 %
	3	318	4 %	NA	0 %
	NA	299	0 %	302	0.5 %
MM19	1	298	53 %	299	38 %
	2	304	4 %	305	4 %
	3	292	33 %	293	55 %
	4	316	2 %	NA	0 %
	5	270	2 %	269	0.5 %
	6	253	3 %	NA	0 %
	7	281	3 %	281	0.5 %
	NA	310	0 %	311	1 %
TP14	1	296	78 %	297	60 %
	2	305	22 %	306	40 %
MS1	1	361	100 %	362	99 %
	NA	327	0 %	326	0.5 %
	NA	384	0 %	386	0.5 %
MS9	1	455	100 %	444	96 %
	NA	443	0 %	432	1.5 %
	NA	461	0 %	450	2 %

DNA was prepared and initially sized at the University of Glasgow (UoG), then amplified and sized at Moredun Research Institute (MRI). In addition the prevalence of each allele is given for the two studies. Only primary peaks were used to assign allele number

^a^Allele sizes are the binned Genescan results

### Accuracy

**Fig. 1:**
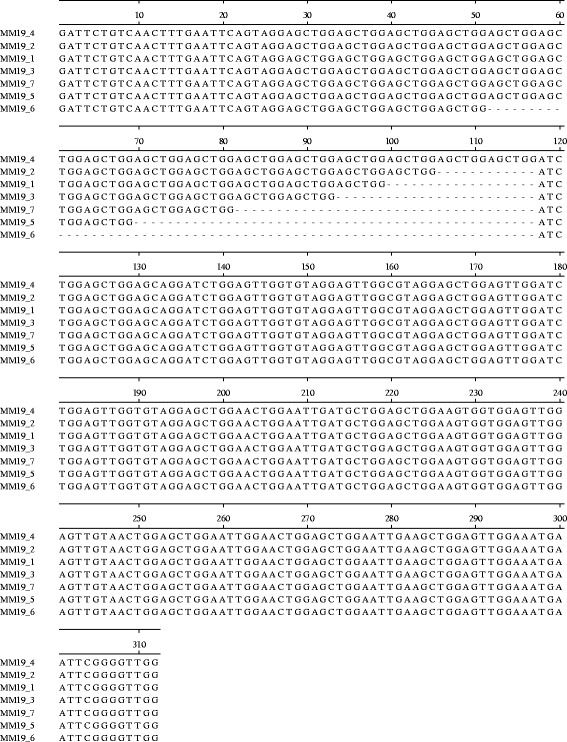
Alignment of sequences of *Cryptosporidium parvum* MM19 alleles, demonstrating the variable number of repeat regions differentiating alleles

Representatives of all identified alleles were subjected to sequence analysis. All sequences analysed showed that the different alleles varied only in the repeat regions (Fig. 1) and that the difference in fragment size corresponded to variable numbers of repeat motifs. As well as aligning with the *C. parvum* reference genome, some of the alleles matched “microsatellite” sequences already deposited in GenBank from similar studies of *C. parvum* [22, 23] with 0–2 base differences: MM5 allele 2 (JX413509), MM18 allele 2 (JX413498), MM19 allele 1 (JX413503), MM19 allele 3 (JX413502), TP14 allele 1 (JF342561) and TP14 allele 2 (JF342562). Where present, base differences were outwith the repeat region and for TP14 and MM19 were within a primer binding site. Sequences of alleles have been deposited in GenBank under accession numbers KP172504-KP172519 (Table 6).

**Table 6:** Sizes of fragments of *Cryptosporidium parvum* DNA obtained using capillary electrophoresis compared with sizes obtained by sequence analysis

Marker	Allele	Size (bp)		Accession number
		Fragment analysis	Sequence analysis	
MM5	1	261.4	260	KP172504
	2	234.6	233	KP172505
MM18	1	288.1	290	KP172506
	2	293.7	296	KP172507
	3	317.5	320	KP172508
MM19	1	297.6	294	KP172509
	2	303.7	300	KP172510
	3	292.0	288	KP172511
	4	316.3	312	KP172512
	5	269.1	264	KP172513
	6	252.0	246	KP172514
	7	280.3	276	KP172515
TP14	1	295.6	301	KP172516
	2	304.3	310	KP172517
MS1	1	361.2	362	KP172518
MS9	1	455.1	450	KP172519

The size of sequenced products differed from those determined by fragment analysis (Table 6); sizes obtained by CE were larger than sizes obtained by sequencing for markers MM5, MM19 and MS9 but sequenced sizes were larger for markers MM18, TP14 and MS1.

### Discriminatory ability

Out of the 6 markers trialled with fragment typing, 2 were found to be monoallelic (MS1 and MS9), and two were biallelic (MM5 and TP14) (Table 3). MM18 had 3 alleles but MM19 was the most polymorphic marker, with 7 alleles identified. MM19 was confirmed as the most discriminatory marker (SID 0.60) followed by *gp60* then MM18 (Table 3).

**Fig. 2:**
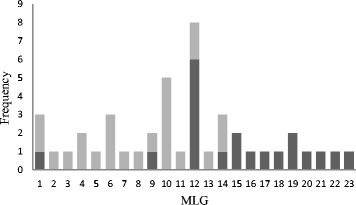
Frequency distribution of multilocus genotypes (MLGs) of *Cryptosporidium parvum* identified in 118 samples from 19 farms in Scotland (light grey) and 20 farms in Cheshire (dark grey)

There was limited evidence of mixed genotype infections within individual calves, with 109/118 calves shedding single alleles at each locus. In addition single MLGs were detected on most farms. Overall 23 MLGs were identified in 118 samples, from 39 different farms in two regions of the UK (Fig. 2). The majority of MLGs (14/23) were only identified on one sampled farm, however some MLGs were more widely distributed with 4 MLGs common to both the Cheshire (2004) and NE Scotland (2011) studies. One MLG was particularly prevalent, having been identified on 8 farms representing both studies (MLG 12).

SID for the MLFT tool was 0.92 (0.90–0.94), compared to 0.44 (0.33–0.54) by *gp60* sequence typing alone. Using the three most informative markers (MM19, *gp60* and MM18) would have differentiated 18/23 MLGs with an overall SID of 0.85 (0.81–0.90). The four most informative markers (MM19, *gp60*, MM18 and TP14) would have differentiated 21/23 MLGs with a SID of 0.89 (0.85–0.92).

## Discussion

As with diagnostic test development, validation of any microbial typing system is essential. Guidelines have been set out for bacterial typing systems [24, 25], and many of these guidelines can be applied to parasites, particularly haploid protozoa such as *Cryptosporidium*. In the current study, performance of MLFT was assessed in bovine-derived *C. parvum* and found to provide good typeability, specificity, precision and discrimination.

Markers were selected as they had shown promising results in other studies of bovine *C. parvum* [12, 22, 26], and were also ranked highly in a recent review of published multilocus genotyping methods [15]. An ideal typing scheme would have markers distributed evenly across several chromosomes [27], which is not the case in the current scheme - of the 4 most discriminatory markers, three are on Chromosome 8. MM18 and MM19 are some distance from each other, although TP14 and MM18 are somewhat closer on Chromosome 8. Selecting markers on different chromosomes would remove any confounding effect of physical linkage and provide the added value of enabling data generated to be analysed robustly at the population genetic level. As more genomic data becomes available for *C. parvum*, it should be possible to select more appropriate markers for MLFT schemes.

Typeability using nested PCR was considered acceptable at 84 %. It was observed that samples which were variably amplified with 18S rRNA primers (for example only amplifying in one or two of three replicates) were also difficult to amplify using MLFT primers (data not shown) and 7 samples failed to amplify with any of the MLFT primers. This suggests that these samples contained low levels of, or poor quality, *C. parvum* DNA, as the 18S rRNA protocol has been shown to be very sensitive, perhaps due to the multi-copy nature of this gene. DNA was prepared from stool using standard methods, however template quality may have adversely affected typeability. The DNA samples from the Cheshire study were prepared in 2004, 10 years before use in the current study, possibly allowing degradation of DNA. In addition, samples may have contained low numbers of oocysts as calves were not sampled on the basis of clinical signs in either study - many calves were not in the acute stage of infection. Typeability obtained with MLFT has been shown to compare favourably with MLST [13], possibly due to the “stutter effect” where tandem repeat units interfere with sequencing.

Although *C. parvum* is the most prevalent species in young calves, *C. bovis* and *C. ryanae* are also occasionally identified, whereas *C. andersoni* is usually found in older animals [7, 28]. We wanted to verify that the primers used would not co-amplify any non-*C. parvum* species in undetected mixed species infections, as this could be misinterpreted as a new (*C. parvum*) allele. The current study trialled a limited number of non-*parvum* species prevalent in bovines and found that the primers did not amplify these species. However, where environmental, human or wildlife samples are to be typed a greater range of species may be identified, therefore primer-BLAST was used and it was established that *C. hominis* could be co-amplified with these primer pairs, as shown in previous studies using these markers [26]. This may be of value to public health laboratories as the same scheme could potentially be used for both of the major causes of cryptosporidiosis in humans. However a comprehensive review of MLFT markers in both species concluded that different sets of markers are probably required for each species [15]. It is always advisable to first assign samples to species level before further typing.

Precision, in terms of the repeatability of sizes obtained within our laboratory, was good, with the possible exception of MM5 allele 2. Between laboratories, fragment sizes did differ to some degree resulting in reduced reproducibility of sizing. The consistent difference of 11 bp in MS9 sizing remains to be explained and unfortunately sequencing data is not available for the historical data. We consider it unlikely that this is a true reproducibility issue, given that it is limited to this marker however MS9 was excluded from further analysis as it was monoallelic in our samples, along with marker MS1. Therefore there are challenges in comparing MLFT results between laboratories unlike, for example, sequence data; however, crucially, our results show that the tool is reproducible with respect to allele assignation. Larger scale inter-laboratory validation is now warranted. In the future it may be beneficial to have marker-specific size standards which include sizes of all known alleles, aiding reproducibility.

The current protocol may not accurately measure size, as demonstrated when sequence and fragment sizes were compared. Again, measuring the size accurately may not be as important as assigning the correct alleles. Sequence and fragment size obtained by CE have been shown to differ in other studies [13]. One aspect that may affect accuracy is the size standard used. ROX400HD has 21 size markers, compared to 16 for ROX500, therefore it is more accurate in sizing fragments up to 400 bp. In addition, Applied Biosystems’ literature states that the marker at 250 bp cannot be used to size samples with ROX500 as it is sensitive to small temperature variations in CE. Fragments in this range may be sized less accurately, which may particularly impact on MM5, with alleles of 235 bp and 262 bp. However, MS9 has fragments >400 bp so ROX400HD could not be used for this marker; for consistency, ROX500 was used throughout.

The level of discrimination required by a particular typing tool depends on the epidemiological question being addressed. The population genetics of the microbe should also be considered. Here we were seeking a tool to answer geographically and temporally local epidemiological questions in a relatively conserved parasite. In *Cryptosporidium*, the ideal MLFT tool should have the discriminatory ability to differentiate geographically local isolates [27], and for this reason we used isolates from two cross-sectional studies, which sampled farms from relatively small spatio-temporal windows. The results show that the typing scheme was able to fulfil this criterion, as 14/23 MLGs were unique to sampled farm although most of the MLGs detected were part of the same clonal complex (data not shown). In addition, the finding that most calves and farms had single MLGs suggests that the scheme is not overly discriminatory for regional (such as catchment-level) studies. An application of this tool is to study transmission dynamics between and within farms, by investigating whether farms have “unique” or “common” MLGs, single or multiple MLGs and to investigate stability over time (manuscript in preparation).

The MLFT scheme showed good discriminatory power when compared to standard subtyping using *gp60* sequencing alone, as demonstrated by SID. This is due to the fact that the majority (85/136) of samples were the common *gp60* subtype, IIaA15G2R1. As samples were not independent but were clustered by farm, the values for SID may not be applicable to the general cattle population - in fact, the non-independence may actually reduce the apparent SID. Some markers were more informative than others - MS1 and MS9 were monoallelic in the samples tested. However, some diversity has been reported with these markers in other studies of cattle [11, 12], although the prevalence of alleles other than those in the current study appear to be very rare in Scottish and Irish calves [12, 22]. Widmer and Sullivan [27] recommended that the minimum number of markers be used to give the required resolution; a recent review estimated that in *C. parvum* there was, on average, 23 % marker redundancy [15]. This is true of the current study, demonstrated by the fact that SID using just the three or four most informative markers was estimated at 85 % (81–90 %) and 89 % (85–92 %) respectively.

Other studies utilising fragment sizing have included a similar region of the GP60 gene to that used in sequence analysis for subtype assignment; when used in this way it is often referred to as GP15. We chose to use sequence analysis for this gene as this method is a good library typing tool, having been adopted almost universally by *Cryptosporidium* researchers worldwide allowing for easy comparison of subtypes. Being sequence based it offers more discrimination than fragment sizing alone but mixed profiles can be problematic. As shown in the current study, the discriminatory ability of this single locus sequence type is not sufficient for local epidemiological questions, such as outbreak investigations.

Where harmonised schemes are being developed to allow source attribution, it should be considered whether markers are informative in *C. parvum* derived from both potential sources of oocysts (livestock, wildlife etc.) and humans; previous studies have used our trialled markers to successfully type both human and bovine-derived *C. parvum* [12].

Three additional markers, MSA, MSD and MSF, were applied to representatives of the 23 MLGs identified in the current study using primers reported in the literature [11]; all 3 of these additional markers were monoallelic in our samples producing fragments of 229 bp, 274 bp and 156 bp respectively (data not shown). These sizes correspond with reported allele sizes for these markers [11]. In the current study only isolates of *gp60* subtype IIa were tested; cattle in other countries including Portugal [29, 30] have been shown to infrequently shed *gp60* allele IId, although this allele has not been reported to date in UK cattle. Human *C. parvum* is most commonly *gp60* allele IIa in the UK [6] but other alleles are also occasionally identified, notably IId and, rarely, IIc [6]. More work would be required to determine the performance of the markers proposed in the current study in non-IIa *gp60* subtypes of *C. parvum*.

Calves from both Cheshire 2004 and NE Scotland 2011 cross-sectional studies appeared to shed the same predominant alleles of the markers used and allele frequency distributions were very similar, suggesting that these alleles are fairly stable in UK calf populations. It is also clear that the same alleles are mainly present in other Scottish studies of calves [12], as demonstrated in Table 5; a few additional alleles were detected, probably due to differences in study design, but in very low numbers. We also assessed available literature where the same markers and primers were used. As previously noted, this was limited by the lack of a coordinated approach to marker selection. In addition allele sizes are not always reported; where they are given, it is not possible to prove definitively that reported alleles are the same due to the previously stated problems with fragment sizing. Authors rarely state if reported sizes are sequence sizes or binned fragment sizes. A study of *C. parvum* in Italian livestock used the same marker combination as the current study, but there were some small variations in (second round) primer sequences (TP14 reverse and MM19 forward) [26]. These primer sequences were also applied to *C. parvum* samples collected from calves in Ireland 2003–2005 [22]. Interestingly in both of these studies, size and frequency of alleles reported for MM5, MM18, TP14 and MS1 were similar to those found in the current study: 94–98 % was 233/260 bp for MM5, 65–95 % was 290/296 bp for MM18, 89–95 % was 300/309 bp for TP14 and 66–99 % was 362 bp for MS1. The amended MM19 forward primer sequence used by Drumo *et al.* and De Waele *et al.* aligns with the published reference genome for *C. parvum* and may be superior to that used in the current study. These differences also account for the small number of base differences detected when we aligned our allele sequences with other microsatellite sequences using BLAST.

As well as the improved typeability of MLFT over MLST as reported by Diaz *et al.* [13], we found MLFT also compares favourably to sequencing in terms of time and cost. Although the use of fluorescently-labelled primers adds to the cost of standard PCR, fragment analysis was economical compared to sequencing at approximately £0.80 (1 EURO, $1.28) per read; this could have been reduced by multiplexing PCR products into one well for sizing, either using different fluorescent labels or ensuring that expected fragment sizes were sufficiently different to allow differentiation. In addition, 10/118 samples had more than one allele at one, or more, markers, which would not have been detected using direct sequence typing. This is similar to the 11.6 % of infections found to be mixed in an Italian study of humans and livestock [26]. In other studies, criteria for assignment of mixed genotypes are unclear, or more stringent. For example one Scottish study defined a sample as mixed if the height of the secondary peak was >10 % of the main peak, possibly explaining why they detected a relatively high prevalence of mixed infections - up to 37 % in Aberdeenshire [12]. The prevalence of mixed infections may also increase with age of animal sampled, as older animals will have been exposed to more sources of oocysts. However the advantages of MLST are that it provides greater discrimination than MLFT and both accuracy and reproducibility are superior, in single genotype infections.

Our preferred software was STRand, as we found that PeakScanner had problems with bleed-through in the event of the product being too strong. This problem can be easily detected and manually corrected using STRand. When comparing results we found minimal variation when the same sample was sized with the two different softwares. Both of these softwares are free to download.

## Conclusions

We found MLFT using markers MM5, MM18, MM19 and TP14 performed well in typing bovine *C. parvum*, in combination with results for sequence analysis of the GP60 gene. It appears that the common alleles in cattle in the UK have been identified, although it is expected that further studies will produce new alleles which should be verified and added to the reference collection.

These markers have also shown to have good typeability and discrimination in human *C. parvum* (personal communication, Chalmers), and have also been used successfully in a water catchment level investigation of contamination of a water supply by *Cryptosporidium* oocysts [31]. Therefore we believe this tool has potential value in source attribution in the event of an outbreak of *C. parvum* in humans. The adoption of standardised primers is to be encouraged, and collaboration particularly at regional and national levels between public health bodies and veterinary researchers is vital. A system for standardising the sizing should be considered, potentially including a centrally-curated panel of all known alleles that can be used to calibrate machines or to be run in parallel with test samples. A standardised nomenclature system perhaps based on the number of repeats may also be helpful [32]. It is likely that advancing technology such as next generation sequencing may supersede this tool; however this is not currently economically or practically feasible, particularly in the timeframe required in the event of an outbreak. The process of developing any integrated scheme will lay the foundations for future collaboration, as long as participants maintain a flexible and open-minded approach to future technological developments.
